# Semaglutide‐Induced Hepatic Injury: A Rare Case of Drug Induced Liver Injury

**DOI:** 10.1002/ccr3.70783

**Published:** 2025-08-06

**Authors:** Rupayan Kundu, Lyudmila Shtoff

**Affiliations:** ^1^ Department of Internal Medicine Cleveland Clinic Foundation Cleveland Ohio USA; ^2^ Department of Internal Medicine Cleveland VA Medical Center Cleveland Ohio USA

**Keywords:** drug‐induced liver injury (DILI), GLP‐1 receptor agonist, hepatotoxicity, liver enzyme elevation, semaglutide

## Abstract

Semaglutide, a glucagon‐like peptide‐1 (GLP‐1) receptor agonist, is widely used for type 2 diabetes mellitus and has demonstrated hepatoprotective effects. However, this case highlights a rare instance of possible drug‐induced liver injury (DILI) temporally linked to its use. A middle‐aged male with well‐controlled diabetes, social alcohol use, and no history of liver disease presented with asymptomatic elevations in alanine transaminase (ALT) and aspartate aminotransferase (AST). Extensive workup, including undetectable blood ethanol level, normal viral hepatitis panel, and unremarkable liver ultrasound, revealed no alternative etiology. Given the temporal association and absence of other factors, semaglutide was suspected as the culprit, leading to its discontinuation. A rapid decline in transaminase levels upon withdrawal supported this diagnosis. Although semaglutide is primarily metabolized through proteolytic cleavage and beta‐oxidation with minimal hepatic involvement, this case suggests the possibility of idiosyncratic liver injury. The mechanism remains unclear but may involve metabolic stress, weight loss, or biliary dysfunction. With the increasing use of semaglutide, clinicians should maintain a high index of suspicion for unexplained liver enzyme elevations, particularly following dose adjustments.

**Summary**

- Although semaglutide is typically hepatoprotective and rarely associated with liver injury, clinicians should remain vigilant for idiosyncratic drug‐induced liver injury (DILI).
- In patients with unexplained liver enzyme elevations during semaglutide initiation or dose increase, prompt discontinuation often leads to rapid normalization, underscoring the importance of monitoring hepatic function closely.

## 1 Introduction

Semaglutide is a synthetic analog of the glucagon‐like peptide‐1 (GLP‐1) receptor agonists, primarily utilized in managing type 2 diabetes mellitus following its approval for injectable use in December 2017 [1]. Although generally well‐tolerated, common adverse effects include hypoglycemia and gastrointestinal disturbances. Nevertheless, more severe complications such as pancreatitis, diabetic retinopathy, and acute kidney injury have been occasionally reported [2]. Hepatobiliary issues linked to semaglutide are uncommon and mainly involve gallstone‐related pathology [3]. Semaglutide is primarily metabolized via proteolytic cleavage and beta‐oxidation, with minimal hepatic involvement, posing a low risk for drug‐induced liver injury (DILI) [4]. In this report, we describe a rare instance of liver injury potentially attributable to semaglutide administration.

## 2 Case History/Examination

A male patient in his early 50s with a medical history significant for Type 2 diabetes mellitus (last HbA1C 6.9%), hypertension, dyslipidemia, 1–2 beers per week alcohol use (last drink 1 week back), presented to the primary care (PCP) visit for routine follow‐up care. His medications included atorvastatin 40 mg, losartan 25 mg daily, empagliflozin 12.5 mg/metformin 1000 mg twice daily, and semaglutide subcutaneous injections weekly, initiated on September 2024 with 0.25 mg subcutaneously weekly, and increased in October 2024 to 0.5 mg; last dose was on the day prior to clinic visit (March 10, 2025). He denied any recent illness or exposure to sick contacts at home. He is sexually active with a single partner and has no concerns for sexually transmitted infections. He also denies the use of herbal products, supplements, or any other over‐the‐counter medications. At the visit, labs revealed elevated alanine transaminase (ALT) and aspartate aminotransferase (AST).

**FIGURE 1:**
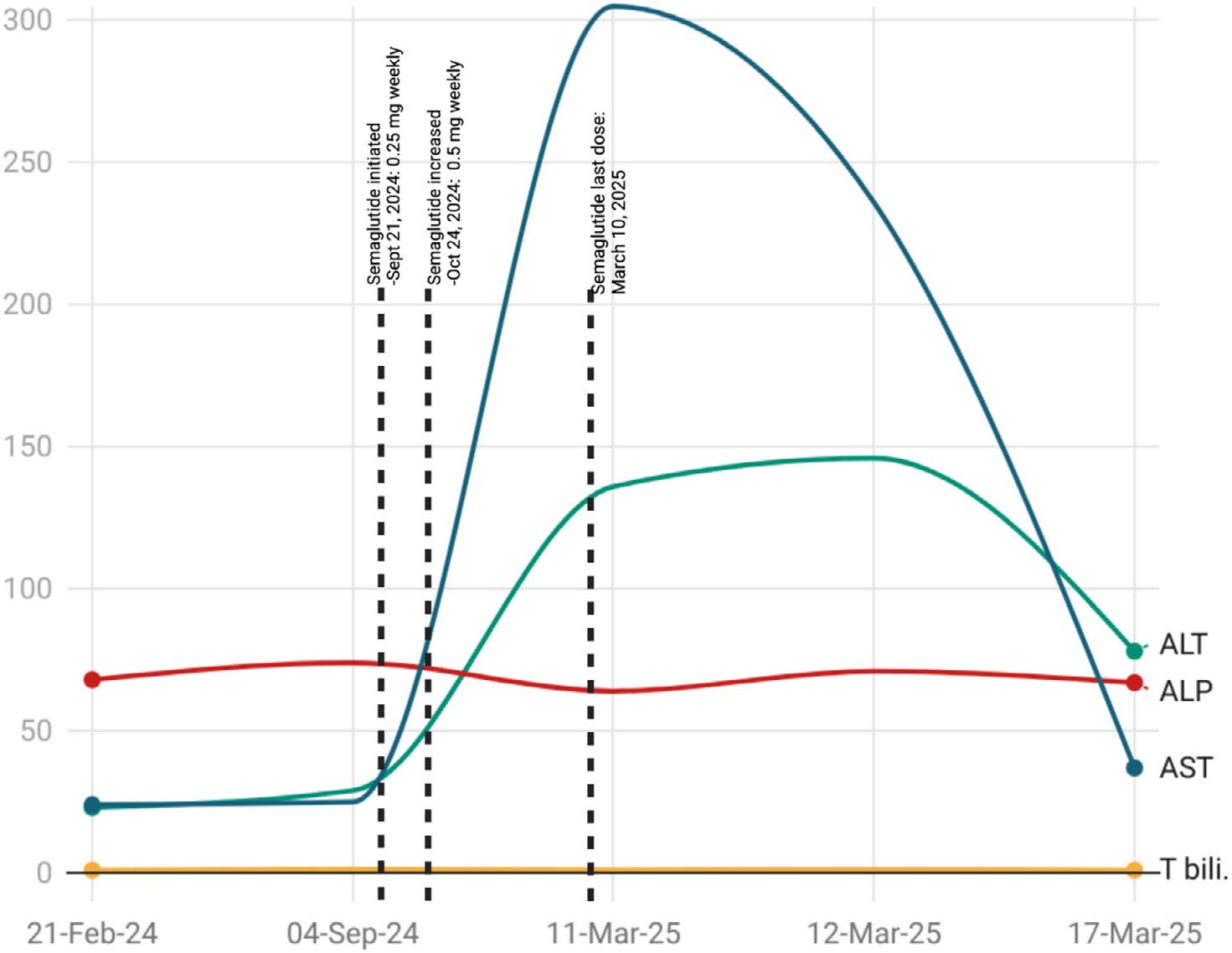
Trends in liver enzymes before, during, and after discontinuation of semaglutide therapy.

The patient was hemodynamically stable and remained entirely asymptomatic, with no reports of jaundice, abdominal discomfort, or systemic symptoms indicative of hepatic dysfunction. Routine lab tests on the PCP visit (March 11, 2025) revealed significantly elevated AST (305 U/L) and ALT (136 U/L). Other liver function parameters, including bilirubin and alkaline phosphatase, as well as ethanol, complete blood count, and kidney function tests, were within normal limits. Lab values are given in Table 1. A repeat test on the next day confirmed persistently elevated AST (236 U/L) and ALT (146 U/L). At his last primary care visit in September 2024, hepatic enzymes were within normal limits. Figure 1 demonstrates the trends in liver enzymes before, during, and after discontinuation of semaglutide therapy. Given this acute elevation, a comprehensive viral hepatitis panel was obtained, which revealed reactive hepatitis B surface antibody and hepatitis A IgG antibody, indicating immunity from prior vaccination or resolved infection. However, the patient tested negative for active hepatitis B surface antigen, hepatitis B core antibodies (IgM and total), hepatitis C antibody, and hepatitis A IgM, effectively ruling out an active viral hepatitis etiology. Liver ultrasound without Doppler revealed partially obscured echogenic liver without biliary obstruction or mass. Notably, the patient has a remote history of NSAID‐induced gastrointestinal ulceration, which precludes the use of NSAIDs in him.

**TABLE 1:** Lab values before, during, and after discontinuation of semaglutide therapy.

Test	September 04, 2024		March 11, 2025	March 12, 2025	March 17, 2025	Reference range
AST (U/L)	25	Semaglutide initiated 0.25 mg weekly in September 2024 and escalated in October 2024 to 0.5 mg weekly, last dose: March 10, 2025	305	236	37	10–40
ALT (U/L)	29		136	146	78	0–55
Alkaline phosphatase (U/L)	74		64	71	67	40–150
Total bilirubin (mg/dL)	1.3		1.1	1.2	1.0	0.2–1.2
Direct bilirubin (mg/dL)	—		—	0.5	0.3	0.0–0.5
WBC (K/cmm)	9.5		—	6.1	—	3.6–11.0
Hemoglobin (g/dL)	16.0		—	15.9	—	13.6–17.4
Platelet count (K/cmm)	198		—	139	—	150–400
Ethanol level (mg/dL)			< 10	< 10		< 9.9

## 3 Differential Diagnosis

With semaglutide being the only medication that had been recently started and increased, and in the absence of recent alcohol consumption with an undetectable ethanol blood level, a presumptive diagnosis of drug‐induced liver injury (DILI) secondary to semaglutide was made. As an immediate intervention, semaglutide was discontinued, and the patient was advised to abstain from alcohol use temporarily. Although autoimmune hepatitis was considered in the differential diagnosis, specific autoantibody testing (e.g., ANA, SMA, LKM1, anti‐SLA/LP) was not pursued due to the rapid normalization of liver enzymes following drug discontinuation and the absence of clinical features suggestive of chronic liver disease.

## 4 Outcome and Follow‐Up

A follow‐up testing 1 week post‐discontinuation revealed a notable improvement in liver enzymes (AST: 37 U/L, ALT: 78 U/L). Given the rapid resolution of hepatic transaminase elevation and unremarkable liver ultrasound without Doppler, further investigations, including liver ultrasound with Doppler and tests for Wilson's disease or hemochromatosis, were deemed unnecessary. Throughout this period, the patient remained clinically well, with no signs of jaundice, abdominal pain, or other indicators of hepatic dysfunction. He was scheduled for regular follow‐up evaluations to monitor liver function and reassess his clinical status.

## 5 Discussion

This case report highlights a rare instance of liver injury temporally associated with semaglutide use, raising the possibility of an idiosyncratic drug‐induced liver injury (DILI) in a middle‐aged male.

Semaglutide is widely recognized for its beneficial effects on metabolic health, including glycemic control, weight loss, and hepatic improvement in non‐alcoholic fatty liver disease (NAFLD) and non‐alcoholic steatohepatitis (NASH). In fact, multiple studies have demonstrated that semaglutide reduces hepatic steatosis, decreases inflammation, and improves overall liver function, making it a promising therapeutic option for metabolic‐associated liver disease [5, 6]. To date, only a few case reports, including one presented as a conference abstract [7], reported semaglutide‐induced liver injury [7, 8], but its effects are generally hepatoprotective rather than hepatotoxic.

The pattern of liver enzyme elevation observed in this patient could mimic that seen with binge alcohol use, but this was excluded based on a thorough clinical history. Although the ethanol level was undetectable, more objective biomarkers such as phosphatidylethanol (PEth) level were not obtained; there was no clinical suspicion of alcohol‐related liver injury. An acute hepatitis panel was negative. Given the rapid resolution of hepatic transaminases, further investigations, including tests for Wilson's disease or hemochromatosis, were deemed unnecessary. We acknowledge that the lack of autoimmune serologies limits our ability to definitively exclude autoimmune hepatitis type 3 or drug‐induced autoimmune‐like hepatitis.

The cause of DILI secondary to semaglutide is not known. While semaglutide is not known to cause direct hepatotoxicity, it has been associated with rapid metabolic changes, including significant weight loss, which may stress hepatic function, particularly in individuals with underlying liver disease. A previous case report described liver decompensation in a patient with NASH‐associated cirrhosis following rapid weight loss induced by semaglutide, highlighting a potential indirect effect on liver function [9]. Additionally, semaglutide has been linked to gallbladder and biliary tract diseases, including cholelithiasis and cholecystitis, which could contribute to hepatic dysfunction in predisposed individuals [8]. However, our patient had neither a history of cirrhosis nor liver disease nor rapid weight loss nor laboratory findings indicative of a cholestatic pattern.

With the recent increase in the use of semaglutide day by day, it remains a highly effective and generally hepatoprotective medication; clinicians should remain vigilant when initiating therapy, particularly in patients with pre‐existing liver disease or those experiencing unexplained liver enzyme elevations.

## Author Contributions

**Rupayan Kundu:** conceptualization, data curation, methodology, supervision, validation, writing – original draft, writing – review and editing. **Lyudmila Shtoff:** supervision, validation, writing – review and editing.

## Consent

Written informed consent obtained from the patient.

## Conflicts of Interest

The authors declare no conflicts of interest.

## Data Availability

The data that support the findings of this study are available on request from the corresponding author. The data are not publicly available due to privacy or ethical restrictions.
